# Leaf Mustard (*Brassica juncea*) Germplasm Resources Showed Diverse Characteristics in Agro-Morphological Traits and Glucosinolate Levels

**DOI:** 10.3390/foods12234374

**Published:** 2023-12-04

**Authors:** Awraris Derbie Assefa, Seong-Hoon Kim, Ho Chul Ko, Nayoung Ro, Parthiban Subramanian, Yun-Jo Chung, Yong-Hyuk Lee, Bum-Soo Hahn, Ju-Hee Rhee

**Affiliations:** 1 National Agrobiodiversity Center, National Institute of Agricultural Sciences, Rural Development Administration, Jeonju 54874, Republic of Korea; awrarisderbie@gmail.com (A.D.A.);; 2 Department of Biotechnology, Debre Berhan University, Debre Berhan P.O. Box 445, Ethiopia; 3 National Creative Research Laboratory for Ca^2+^ Signaling Network, Jeonbuk National University Medical School, Jeonju 54896, Republic of Korea; yjchong@jbnu.ac.kr; 4 Agricultural Technology Center of Yeosu, Yeosu 59633, Republic of Korea

**Keywords:** agro-morphological characters, diversity, glucosinolates, leaf mustard

## Abstract

Leaf mustard, characterized by its purple/red/green leaves with a green/white midrib, is known for its thick, tender, and spicy leaves with a unique taste and flavor. There were only a few studies reported on leaf mustard for its morphological and biochemical traits from Korea. A total of 355 leaf mustard accessions stored at the GenBank of the National Agrobiodiversity Center were evaluated for 25 agro-morphological traits and seven intact glucosinolates (GSLs). The accessions showed a wide variation in terms of most of the traits. The quantitative agro-morphological traits varied from 16.0 (leaf length) to 48.7% (petiole width) of the coefficient of variation (CV). The highest variation was observed in glucoiberin (299.5%, CV), while the total GSL showed a CV of 66.1%. Sinigrin, followed by gluconapin and gluconasturtiin, was the most abundant GSL, accounting for as high as 75% of the total GSLs, while glucobrassicanapin and glucoiberin were the least abundant, contributing 0.7% and 0.1% on average, respectively. Sinigrin had a positive significant correlation with all GSLs but gluconasturtiin, while glucobarbarin and gluconasturtiin were highly positively correlated to each other, but least correlated with other GSLs. The leaf length was negatively correlated with sinigrin and glucoiberin. The width of the petiole showed a positive correlation with gluconapin, glucobrassicanapin, and glucobrassicin, while the length of the petiole had a negative correlation with sinigrin, glucobrassicanapin, glucoiberin, glucobrassicin, and the total GSLs. A higher width of the midrib was associated with higher contents of gluconapin, glucobrassicanapin, and glucobrassicin. A PCA analysis based on the agro-morphological traits showed that the first and second principal components accounted for 65.2% of the overall variability. Accessions that form a head tend to exhibit a longer leaf length, a larger plant weight, a thicker midrib, and higher widths of the midrib, petiole, and leaf. The GSLs showed inconsistent inter-and intra-leaf variation. Accessions that identified for various traits in their performance, such as, for example, Yeosu66 and IT259487 (highest total glucosinolates) and IT228984 (highest plant weight), would be promising lines for developing new varieties.

## 1. Introduction

Mustard (*Brassica juncea*) is an economically important vegetable globally used as an oil seed, vegetable, and condiment with a long history dating back to 3000 B.C. [1]. The most cultivated mustard species are brown mustard (*B. juncea*), black mustard (*B. nigra*), and white/yellow mustard (*Sinapis alba*) [1,2]. Leaf mustard (*B. juncea*, 2n = 36, AABB), ‘gat’ in Korean, is a diploid species of the Cruciferous family produced via hybridization and chromosome doubling of *B. rapa* (2n = 20, AA) and *B. nigra* (2n = 16, BB) [3]. It is characterized by its vigorously growing leaves of a deep purple/red/green color with a green/white midrib. Known for its high resistance to heat, moisture, and cold stress, depending on variety, it is commonly harvested 35 to 40 days after sowing in the summer and 60 to 70 days in the autumn–winter season. Korean mustard has thick, tender, and spicy leaves with a unique taste, aroma, and flavor; hence, it is not only used as a spice but also as the main ingredient in ‘gat-kimchi’ (leaf mustard kimchi) [4]. Mustard leaves help prolong the storage period of ‘gat-kimchi’ due to their slow fermentation speed and maintain a stable color during storage, which could be related to the glucosinolate degradation products it accumulated, which are known for their antimicrobial and anti-fungal effects [4,5].

Glucosinolates (GSLs) are well-known secondary metabolites found in the whole order of Brassicales. GSLs can be found in the roots, seeds, leaf, and stem of the Brassica plant, usually with the youngest tissues containing the highest amount [6]. Florets contain higher concentrations compared to stalks [7]. The content of glucosinolate in *Brassica* generally accounts for about 1% of the dry weight in vegetables and can exceed 10% in the seeds of some plants [8,9]. In general, reproductive tissues (florets/flowers/seeds) contain as much as 10 to 40 times higher glucosinolates than vegetative tissues [10]. The predominant glucosinolates include, but are not limited to, sinigrin (allyl-GS) in mustards and horseradish [11], glucoraphanin in broccoli and cabbage [12,13], glucobrassicin in Brussels sprouts [14,15], gluconapin in Chinese kale shoots [16], glucoiberin in cabbage leaves [14,17,18], glucobrassicanapin in pak choi leaves [19] and kimchi cabbage [20], glucomoringin in moringa leaves [8,21,22], glucoraphasatin in radish roots [23], and gluconasturtiin in watercress leaves [24].

Various biochemical and crop improvement research-related reports are available in the literature. One of the major findings of genetic variability studies of *B. juncea* is delineating the existence of two major gene pools: the Indian and the East European [25]. Pradhan and Pental (2011) [25] have summarized some of the findings before 2007 in genetic divergence using agro-morphology traits, phylogenetic analysis, random amplified polymorphic DNA (RAPD) markers, amplified fragment length polymorphism (AFLP) markers, and biochemicals. In recent years, several studies using agro-morphological characters, SSR molecular markers, and RAPD markers showed a considerable variability among the genotypes of Indian mustard [26,27,28,29,30,31,32,33]. Previous studies of genetic variability among *B. juncea* germplasm collections based on the levels of glucosinolates were focused on the seeds [34,35,36,37], except for Kim et al. (2016) [38] who studied the levels of desulfo-glucosinolates contents in 210 accessions of Korean leaf mustard (*Brassica juncea* var. *integrifolia*). Park et al. (2007) [3] also studied the sinigrin levels of 24 dolsan mustard cultivars and found that the contents varied between 2.07 and 4.38 mg/g. In earlier studies, the aims of breeding programs for Brassica crops were mostly to decrease the level of glucosinolates and increase the oil content [39]. However, in recent years there is a renewed interest in GSLs due to their biocidal and anticarcinogenic effect; hence, increasing the levels of GSLs should also be given attention. Studies on the diversity and relationship among *Brassica juncea* germplasm based on morphological characters, specifically on leaf mustard from Korea, are also not readily available in the literature. Park et al. (2007) [3] analyzed 21 morphological characters in 24 dolsan leaf mustard cultivars and reported a significant variation in the fresh weight, dry weight, leaf length, and the number of leaves.

This study aimed to examine similarities and differences regarding twenty-five agro-morphological characters and seven glucosinolate levels in the leaves of mustard (*Brassica juncea*) germplasm collections in the National Agrobiodiversity Center GenBank. We also studied the relationship between the agro-morphological characters and the individual glucosinolates among the leaves of the genetic resources. This study represented one of the largest sets of *Brassica juncea* samples in a single study and could provide important agro-morphological and biochemical data for further studies. Quantitative data on the glucosinolate contents of Korean mustard leaves using a large population of samples are elusive. Hence, our results provide critical data for mustard consumers and breeders developing new varieties with enhanced levels of bioactive compounds having health-beneficial properties.

## 2. Materials and Methods

### 2.1. Plant Materials

**Figure 1:**
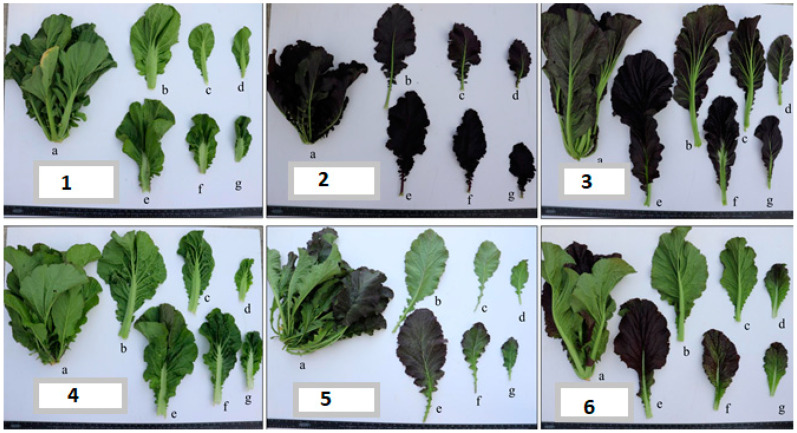
Representative photos of *Brassica juncea* plants (**1**)–(**6**) showing the whole plant (a); the abaxial (b–d) and adaxial (e–g) leaf surfaces; and the outer (b,e), middle (c,f), and inner (d,g) leaves.

The seeds of the leaf mustard plants used in this study were collected from Korea and stored at the Korean GenBank located in the National Agrobiodiversity Center (NAC), Jeonju, Republic of Korea. They were grown in the research farm of NAC in Jeonju (35°49′18″ N 127°08′56″ E). The seeds were sown in plug trays, and the seedlings were grown inside a greenhouse. After 20 days, the healthy-looking seedlings (four to five leaves) were transplanted to an area of 60 × 40 cm per plant in an experimental field. Plant cultural practices were followed as per the recommendation of the Rural Development Administration (RDA) of South Korea. Fertilizers (N-K-P-Ca-B = 65-45-100-100-1.5 kg/10 a) were applied before transplanting the seedlings following RDA standards, and drip irrigation tape was used for watering. A total of 355 accessions were considered in this study. Each accession consisted of 25 plants. Plant growth was maintained using nutrient solutions throughout the growing season. Figure 1 shows some selected representative samples showing the whole plant and the inner, outer, and middle leaves of the *Brassica juncea* plant.

### 2.2. Reagents and Standard Chemicals

The chemicals and solvents used for extraction and analysis were of analytical grade and purchased from Fisher Scientific Korea Ltd. (Seoul, Republic of Korea) and Sigma-Aldrich (St. Louis, MO, USA). Standards of sinigrin, gluconapin, glucobrassicanapin, glucoiberin, gluconasturtiin, glucobarbarin, and glucobrassicin were purchased from Phytoplan Diehm & Neuberger GmBH (Heidelberg, Germany). All standards of glucosinolates were of purity greater or equal to 97%.

### 2.3. Agro-Morphological Characters

Twenty-five agro-morphological characters were investigated based on physical observations using a ruler or a digital scale according to the location, either in the field or in the laboratory. The characters were evaluated based on the International Union for the Protection of New Varieties of Plants (UPOV) descriptors for *Brassica juncea* L. Czern [40]. Leaf length, leaf width, midrib width, midrib thickness, petiole length, and petiole width were evaluated using five matured leaves of randomly selected different plants of the same accession and the average values were reported. The number of leaves and fresh plant weight are reported as the average of four in five plants of the same accession.

### 2.4. Sample Pretreatment and UPLC-MS/MS Analysis of Intact GSLs

Ten to fifteen healthy plants were used for the analysis of GSLs. Leaves were collected from the outer, middle, and inner locations of each plant and mixed. In each accession, three replicate samples were prepared. Great care was taken to prevent the thawing of the sample to minimize the enzymatic degradation of the GSLs. The samples were immediately frozen and all the equipment in contact with them was held at subzero temperatures until further processing. The spatial distribution and inter-leaf variation of the GSLs in *Brassica juncea* were studied using 15 randomly selected genetic resources. The inner, middle, and outer leaves were manually separated for the inter-leaf variation study. Each leaf was then dissected into the top, middle, bottom, and midrib parts, as required. Triplicate samples were prepared for each part.

The samples were processed and extraction was conducted based on a previously reported protocol on *Brassica* spp. [20], with a slight modification. Briefly, the harvested leaves were placed in vinyl and stored in an −80 °C freezer, followed by lyophilization for 48 h using an LP500 vacuum freeze-drier (Ilshinbiobase Co., Seoul, Republic of Korea). The freeze-dried samples were powdered and extracted as follows: 0.1 g ground sample was mixed with 1 mL of CH_3_OH/H_2_O (4:1, *v*/*v*) mixture in an e-tube, vortexed (10 min, 30 °C), and centrifuged (13,300 RPM, 4 °C, 10 min) using a VS-180 CFi centrifuge (Vision Scientific Co., Daejeon, Korea). The supernatant was transferred to an LC vial and the GSLs were analyzed immediately using an UPLC-MS/MS.

Qualitative and quantitative analyses of the GSLs were conducted using the ultra-performance liquid chromatography-tandem quadrupole mass spectrometry Acquity UPLC System (Waters, Milford, MA, USA) and the Xevo™ TQ-S system (Waters, MS Technologies, Manchester, UK). Separation was carried out using an Acquity UPLC BEH C18 (2.1 × 100 mm Id, 1.7 μm,) column (Waters Corp., Manchester, UK). The separation conditions were set as follows: a flow rate of 0.5 mL/min; a column temperature of 35 °C; an injection volume of 5 μL; and a sampling chamber kept at 6 °C. The mobile phase compositions were 0.1% of TFA in H_2_O (A) and 0.1% of TFA in MeOH (B). The elution timetable was set as follows: the initial condition set at 100% of A; 0.0–1.0 min, 100% of A; 1.0–7.0 min, from 100 to 80% of A; 7.0–10 min, from 80 to 0% of A; 10–11 min, from 0 to 100% of A; 11–15 min, 100% of A. The MS/MS analysis was conducted in a negative ion electrospray ionization (ESI-) and multiple reaction monitoring (MRM) mode. The ionization source parameters were set as follows: capillary and con voltages of 3000 V and 54 V, respectively; ion source and desolvation temperatures of 150 °C and 350 °C, respectively; and cone and desolvation gas flow rates of 150 L/h and 650 L/h, respectively. Data acquisition was performed using the MassLynx 4.1 software. The concentrations of individual GSLs were calculated using linear regression equations obtained from calibration curves constructed using authentic standards. The results were calculated from the peak area responses and presented as μmol/kg of the sample dry weight (DW). The MRM transitions and other analysis parameters are presented in Table 1.

### 2.5. Statistical Analysis

The samples were prepared in biological triplicates and all the data presented are means of the replicate measurements. Pearson correlation and Duncan post hoc tests were performed using SPSS V25.0 (Chicago, IL, USA). The quantitative agro-morphological traits and GSLs contents in *Brassica juncea* were analyzed using principal component analysis (PCA) with SIMCA v. 13.0.3 (Umetrics, Umea, Sweden) without preprocessing. The data were visualized using score and loading plots of the first and second principal components. Each solid triangle on the score plot and the loading plot represented an individual sample and the contribution of an individual trait to the score, respectively.

## 3. Results

### 3.1. Variations in Agro-Morphological Traits among 355 Brassica juncea Accessions

Qualitative agro-morphological characters including plant habit (posture), head formation, leaf attitude at the apical part, leaf type, leaf shape, leaf waxiness, lobation of leaf margins, density of incisions of margins, size of the terminal lobe, number of serrates, leaf anthocyanin coloration, blistering, pubescence, and midrib transection were analyzed for 355 accessions based on physical observations in the experimental field and laboratory. Detailed information about the qualitative morphological characters of the genetic resources is presented in Table 2. The characters were evaluated based on the International Union for the Protection of New Varieties of Plants (UPOV) descriptors for *Brassica juncea* L. Czern (UPOV, 2017).

Quantitative agro-morphological traits including the petiole (length and width), leaf (length and width), midrib (width and thickness), plant weight, and the number of leaves per plant were measured using a ruler and a digital scale accordingly. Wide variabilities were recorded among the 355 accessions. The leaf length and width ranged from 20.8 to 57.0 cm and from 8.6 to 31.8 cm, with mean values of 36.3 and 18.1 cm, respectively. The length of the petiole ranged between 0.9 and 12.5 cm with an average value of 3.1 cm, while its width ranged between 0.4 and 3.8 cm with an average value of 1.3 cm. The thickness and width of the midrib also exhibited a broad range of 0.27 to 1.05 cm and 0.6 to 3.8 cm, respectively. An individual plant weighed from 91.7 to 1854.3 g of fresh weight, with an average weight of 338.9 g. A single *Brassica juncea* plant considered in this study could bear anything between 6 to 28 leaves and 16.3 leaves on average. The presence of intra-specific variations is a desirable condition for breeding programs. A wide range of variabilities for different leaf agro-morphological characters in *Brassica juncea* has been reported in earlier studies [41,42]. Similar observations were also found in Dolsan leaf mustard cultivars from Korea [3]. An analysis of variance showed highly significant variations for all the agro-morphological characters studied, revealing the presence of considerable genetic variation among the genotypes (Table 3). According to the UPOV, the seed color, the density of the incisions of the margin of the leaves, the blistering of the leaves, and plant head formation are agreed as useful grouping characteristics for examining the distinctness of *Brassica juncea* varieties.

### 3.2. Identification and Quantification of Individual GSLs in Brassica juncea

**Figure 2:**
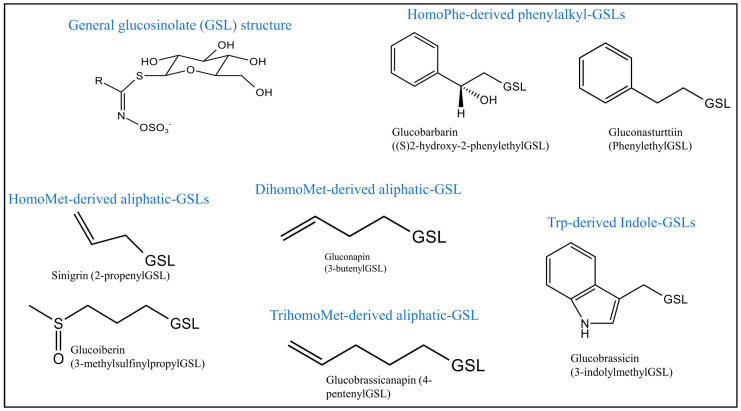

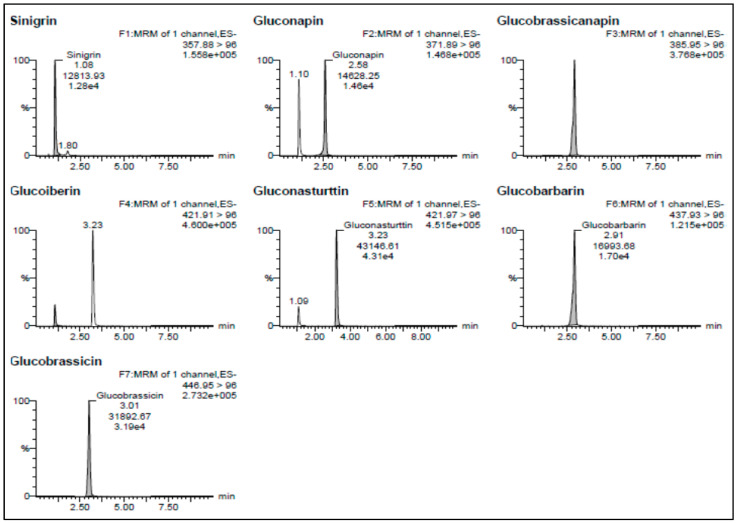
Structure of glucosinolates (**top**) and representative MRM chromatograms of glucosinolates (**bottom**) identified and quantified in *Brassica juncea* accessions.

Seven GSLs were identified in *B. juncea* genotypes and quantified using UPLC/MS/MS (Figure 2). A wide range of variations of the GSLs was recorded among the 355 accessions, in which the total glucosinolates content ranged from 320.4 to 8055.3 μmole/kg DW. The aliphatic glucosinolates (SIN, GNA, GBN, and GIB) were predominant throughout the collections, on average representing 67.3% (22.7~98.3%) of the total GSLs, followed by the phenylakyl (GNS and GBB) and indole (GBS) GSLs, contributing 28.9% and 4.0% to the total GSLs, respectively. Among individual glucosinolates, SIN, GNA, and GNS were the most dominant GSLs, with average contents of 635.9, 496.4, and 401.9 μmole/kg DW, respectively. GIB was the least dominant GSLs, representing between 0.0 and 8.2% of the total GSLs throughout the genetic resources investigated.

**Figure 3:**
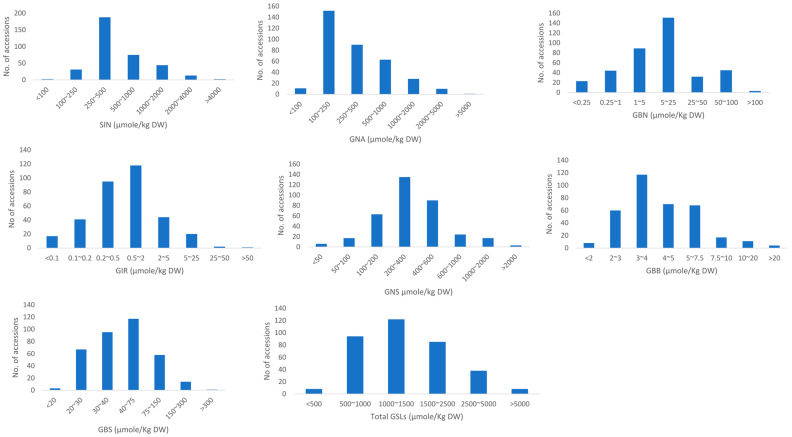
Frequency distribution of individual and total GSLs levels in 355 accessions.

SIN was found to be the most dominant GSL in 148 (41.7%) of the accessions, and its content ranged from 13.0 to 4184.6 μmole/kg DW, accounting for as high as 75% of the total GSLs. Most of the accessions (296) contained less than 1000 μmole/kg DW, while 57 of them accumulated in the range between 1000 and 4000 μmole/kg DW (Figure 3). Two accessions, IT259503 and IT237840, had 4078.7 and 4184.6 μmole/kg DW of SIN contents, respectively. The SIN content varied highly among the genotypes, with a 92.2% coefficient of variation (Table 3). Another aliphatic glucosinolate that was detected in significant levels was GNA, which was the most dominant in 82 of the accessions. GNA ranged between 44.1 and 7708.7 μmole/kg DW, with an average content of 496.4 μmole/kg DW. It was the most varied dominant GSL, with a 126.6% coefficient of variation. However, about 97% of the resources contained less than 2000 μmole/kg DW, while only 2.8% of the accessions had between 2000 and 5000 μmole/kg DW. Yeosu66 had, outstandingly, the highest GNA content (7708.7 μmole/kg DW). The remaining aliphatic GSLs, GBN and GIB, were found in relatively low amounts and comprised 0.0 to 4.8% (0.7% on average) and 0.0 to 8.2% (0.1% on average) of the total glucosinolates, respectively. Although reports on the contents of intact glucosinolates in the leaves of large sets of *Brassica juncea* genetic resources are elusive, some studies from seeds showed SIN and GNA as the predominant GSLs [34,37]. Kim et al. (2016) [38] reported nine desulfo-glucosinolates from the leaves of Korean mustard and found SIN and glucoiberverin as the first and second dominant GSLs in 210 accessions.

GNS, which is considered one of the most widely distributed phenylakyl GSL and is highly dominant in watercress leaves [24], was also found abundantly in our samples, dominating in 125 (35.2%) of the accessions, with values ranging between 27.2 and 3393.5 μmole/kg DW. Most of the accessions (~81%) contained 100 to 600 μmole/kg DW of GNS, followed by 24 accessions accumulating between 600 and 1000 μmole/kg DW, and 19 accessions had between 1000 and 3000 μmole/kg DW of GNS. The highest GNS content (3393.5 μmole/kg DW) was recorded in IT236761. Like SIN, GNS also varied highly, with a standard deviation of 342.9 (coefficient of variation 85.3%). GBB was the other phenylakyl GSL detected in our samples in a relatively low amount, and it ranged from 0.7 to 33.9 μmole/kg DW. The only indole glucosinolate detected in our samples was the Trp-derived GBS. The GBS content varied from 17.3 to 351.9 μmole/kg DW, with an average concentration of 57.9 μmole/kg DW. GBS is known for its health-beneficial effects such as its anticancer activity [43].

### 3.3. Correlation Analysis among GSLs and Agro-Morphological Characters

To investigate the patterns of accumulation of individual glucosinolates and their relationship with quantitative morphological characters, a Pearson correlation analysis was performed using the SPSS software (V25), and the results are presented in Table 4. Among the traits, significant and positive/negative associations were found for some pairs, and some others were uncorrelated. Individual glucosinolates were correlated to each other differently. The highest correlated glucosinolate was SIN, which was significantly (positively) correlated with all but GNS. The two phenylalkyl GSLs, GBB and GNS, were least correlated with all other GSLs but moderately, positively correlated to each other (r = 0.565**). This could be because of the same precursor amino acid (HomoPhe) they both share. As seen in Table 4, the aliphatic GSLs, namely SIN, GNA, GBN, and GIB, were significantly correlated to each other. This could also be attributed to their similarity in their biosynthetic pathway. The leaf length was negatively correlated with SIN and GIB. The length and width of the petiole were correlated with GSLs differently. The width of the petiole showed a positive correlation with GNA, GBN, and GBS, while the length of the petiole had a negative correlation with SIN, GBN, GIB, GBS, and the total GSLs. In addition, a higher width of the midrib was associated with higher contents of GNA, GBN, and GBS. This is in concordance with a previous report where the midrib (white section) accumulates a higher GSLs content than the green part of the leaf of *Brassica rapa* [20]. Correlation studies of the morphological traits and glucosinolates content in *Brassica juncea* are elusive in the literature. In a study comprising 99 accessions of Ethiopian mustard (*Brassica carinata* L.), the SIN content was found to be negatively correlated with most morphological characters investigated, namely, leaf area, leaf length, leaf width, number of primary branches, and plant height, and positively correlated with the length of the petiole [42].

### 3.4. Relationship between the Morphological Characters and the Levels of GSLs

The relationship between some morphological characters and GSLs were evaluated using Duncan’s test (Table 5). Among the characters studied, plant habit (posture), head formation, leaf type, lobation of leaf margins, density of incisions of the margins, leaf waxiness, pubescence on the lower side of the leaf, midrib transection, and the number of leaf serrates had shown significant variation between accessions in groups of distinct agro-morphological characters either on some individual glucosinolates or on the total GSLs. For example, in terms of plant habit, the horizontal types had significantly higher average total GSLs than the semi-erect mustard leaves. The mustard leaves of the division type were significantly lower in their mean total GSLs content than those of the entire (with broad midrib) type. On the other hand, leaf types such as lyrate vs. division, lyrate vs. entire, division vs. lyrate, and division vs. entire (narrow midrib) were not significantly different from each other in their total GSLs content. The average total GSLs content of entire type mustard leaves that had a broad midrib was the highest compared to any of the leaf types (Table 5). This indicates that the midrib of the mustard leaves accumulated higher levels of GSLs, and it agrees with a previous report on other Brassica species [20]. Regarding the midrib transection, the average contents of SIN, GNA, GBN, GBS, and total GSLs were significantly higher in the horizontal compared to the intermediates and semi-circled types. The lobation of the leaf margins had also affected the levels of total GSLs significantly, where the dissected leaves accumulated fewer GSLs compared to the parted leaves. In general, GBS was found to be the most affected chemical with respect to the morphology of the mustard leaves, which exhibited significant variations in eight of the characteristics.

### 3.5. Principal Component Analysis (PCA) Based on Some Agro-Morphological Traits

**Figure 4:**
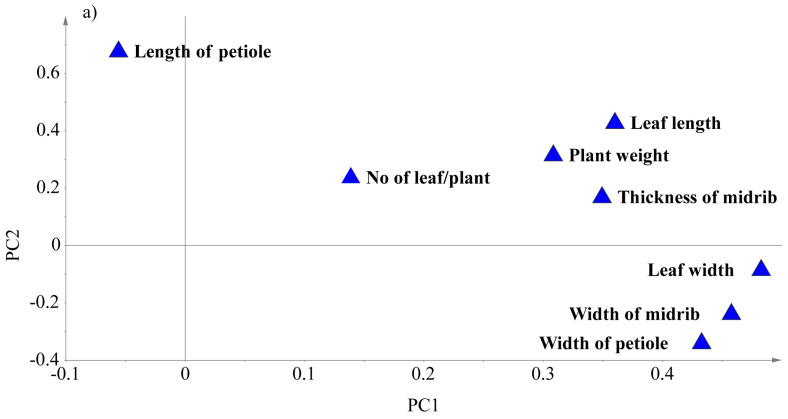

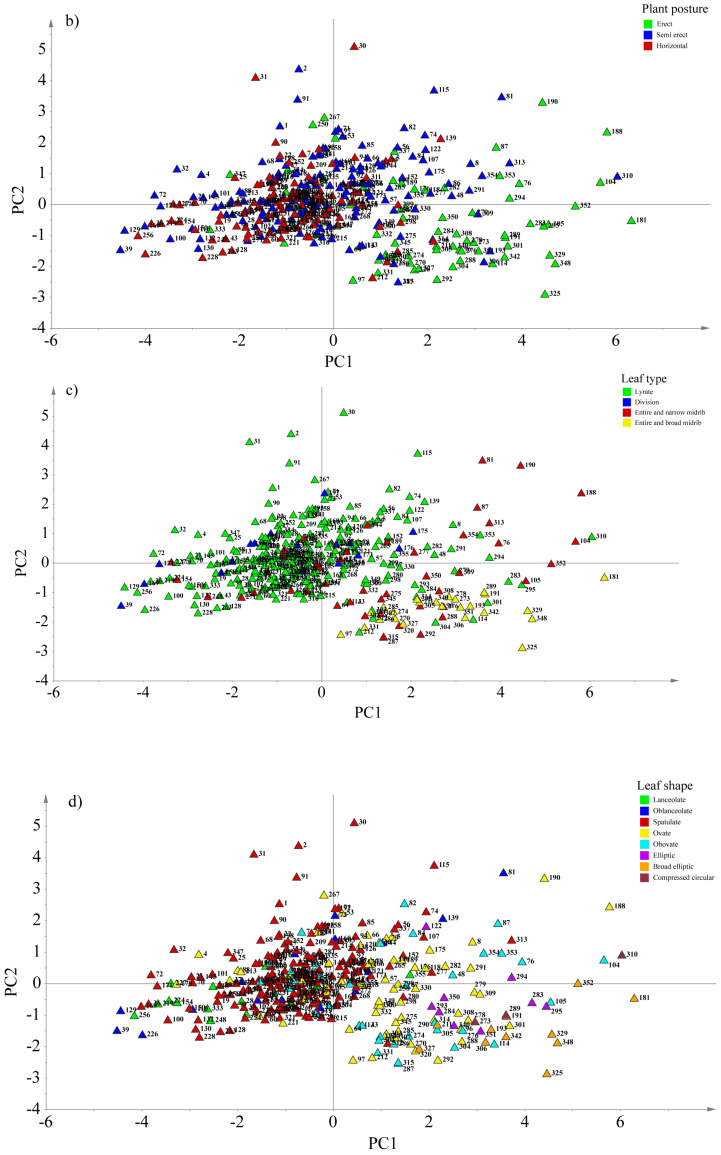

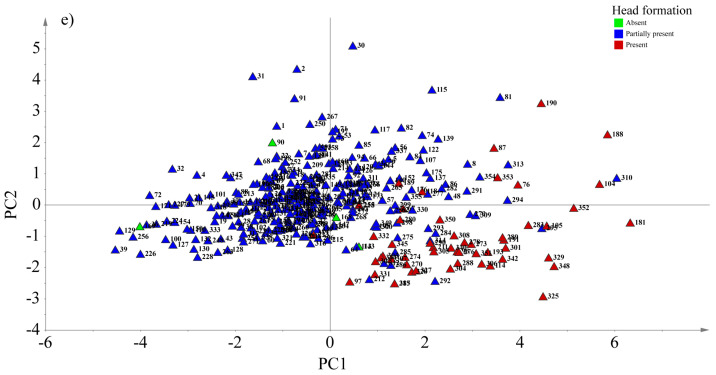
Loading (**a**) and score plots (**b**–**e**) of *B. juncea* samples based on quantitative agro-morphological characters. The score plots are grouped using leaf type, plant posture (**b**), leaf type (**c**), leaf shape (**d**), and head formation (**e**). PC1 and PC2 explained 47.2% and 17.8% of the variations, respectively.

Unsupervised PCA analyses were conducted to identify the prevalence of clusters of genotypes and the most relevant agro-morphological traits. The PCA was carried out based on eight quantitative agro-morphological characters. The first three principal components with Eigenvalues of 3.7, 1.43, and 0.99 accounted for 47.3, 17.9, and 12.5% of the overall variability, respectively. All the traits, but the petiole length (−0.0555), contributed positively to PC1. On the other hand, petiole width (−0.3438), midrib width (−0.2427), and leaf width (−0.0884) contributed negatively to PC2, whereas all the other characters had positive contributions, as shown in Table 6. For PC3, the highest positive and negative contributions were due to the number of leaves (0.9022) and the length of the petiole (−0.2568), respectively. As seen in Figure 4a–e, some genetic resources tend to associate based on specific agro-morphological characteristics such as plant habit, head formation, leaf shape, and leaf type. For example, in terms of the plant growth habit, most erect mustard accessions were distributed on the positive side of PC1, while the horizontal types were on the negative side of PC1 (Figure 4b). The erect types were characterized by a higher petiole width, higher leaf and midrib width, a thicker midrib, a longer leaf length, and a larger plant weight. However, mustard leaves with longer petioles are related to semi-erect and horizontal plant habits (posture). Most of the mustard accessions with longer leaves, wider midrib, leaf, and petiole, and a thicker midrib tended to group at the positive side of the PC1 and were characterized by the entire leaf type as opposed to the lyrate and division types (Figure 4c). The spatulate and lanceolate mustard leaf shapes were characterized by shorter leaf width, midrib width, and petiole width, a thinner midrib, a shorter leaf length, as well as a smaller plant weight. Whereas, circular (compressed), broadly elliptic, and elliptic leaves are characterized by higher widths of the leaf, midrib, and petiole, a thicker midrib, a longer leaf length, and a larger plant weight (Figure 4d). Mustard accessions that form head tend to exhibit a longer leaf length, a larger plant weight, a thicker midrib, and higher widths of the midrib, petiole, and leaf (Figure 4e).

### 3.6. Intra- and Inter-Leaf Distribution of GSLs in Brassica juncea

**Figure 5:**
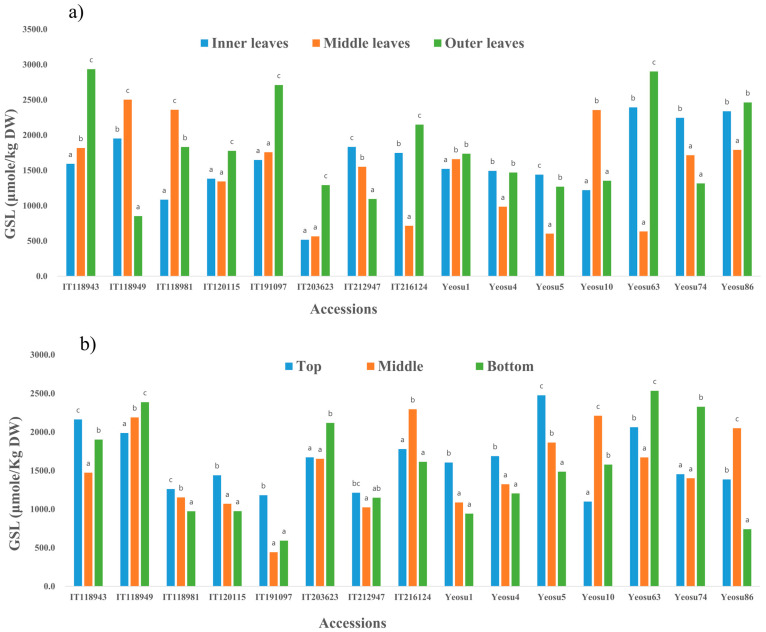
Inter-leaf (**a**) and intra-leaf (**b**) distribution of GSLs in 15 accessions of *B. juncea*. The letters in the graph indicate the differences between the different results.

Fifteen randomly selected light green, green, light purple, and dark purple colored accessions were used to study the spatial distribution of glucosinolates in mustard leaves. To study the distribution of GSLs based on their location/development, the leaves were sampled based on their location (development) as inner (young), middle, and outer (old). On the other hand, to study the spatial distribution pattern within the leaf, the outer part (older) leaves were separated into the top, middle, bottom, and midrib sections. The distribution of total glucosinolate levels based on the leaf sections and position in the plant are presented in Figure 5. There was a significant variation in the GSLs levels between different layers (inner, middle, outer) of leaf samples within the same plant and between the sections (top, middle, and bottom) within the outer leaves. The total GSL level was significantly higher in the outer layers of the leaves (Figure 5a) in 53% of the samples, while the inner and middle layers had significantly higher GSLs in 20% of the samples each. Contrary to this report, the younger leaves (inner leaves) of B. oleracea var. capitate and Raphanus sativus were shown to exhibit higher GSLs levels compared to the older leaves (outer leaves) [44,45]. On the other hand, the top section of the leaves had significantly high GSLs in 47% of the samples, whereas approximately 40% of the samples had significantly higher glucosinolates in their middle and bottom leaf sections (Figure 5b). However, the trend in the total GSLs content based on neither the leaf layers (inner, middle, outer) nor the sections (top, middle, and bottom) of the outer leaves was strictly consistent. A similar observation has been observed in a study employing three samples of kimchi cabbage [20].

## 4. Discussion

In this study, we employed 355 *Brassica juncea* accessions stored at the GenBank of the National Agrobiodiversity Center to study the variations on 25 agro-morphological characters and seven intact glucosinolates. The accessions showed wide variations in terms of both their agro-morphological traits and their GSLs levels. Significant correlations between the traits were also observed. We also performed an unsupervised PCA to identify the prevalence of clusters of accessions based on some selected agro-morphological traits. Finally, based on their quantitative trait performance, we identified potential candidate accessions for further studies.

The *Brassica juncea* accessions showed a huge variation regarding their agro-morphological characters. A different range of variability within and between the leaves has been observed for various agro-morphological characters including plant weight, number of leaf lobes, leaf length, leaf width, number of leaves per plant, anthocyanin coloration, petiole length, leaf shape, and number of serrates [3,29,42,46,47,48,49]. The variability in the agro-morphological characteristics observed in our study is in concordance with some of the previous observations. In addition to the agro-morphological traits of the leaves, previous reports had also explored the diversity in other parts of mustard plants [29,47,48,49], in their physiological traits, and in their biochemical traits such as their antioxidant activities and the total soluble sugars [46]. Rabbani et al. (1998) [49] investigated various morphological traits in the seedling, flowering, and maturity stages of 52 accessions of *Brassica juncea* collected from Pakistan and found narrow phenotypic variations amongst them. The authors highlighted that the reason could be the narrow genetic base of the germplasms that have undergone a high level of genetic erosion. In another multivariate agro-morphological study using the leaf, flower, siliqua, stem, seed, and phenological characters of Indian mustard, 62 accessions showed four distinct groups of varieties based on geographical location, with leaf, stem, and phenological characteristics accounting for much of the variability [48]. The diverse characteristics in the agro-morphology along with the biochemical contents of *Brassica juncea* plants offer opportunities for consumption purposes and the development of improved varieties.

Seven individual glucosinolates were detected in the leaves of mustard accessions. Sinigrin, followed by gluconapin and gluconasturtiin, was the most abundant glucosinolate in most of the accessions. Our results were consistent with previous studies on germplasm collections of leaves [38] and seeds [37] of *Brassica juncea*. The number of glucosinolates detected in our study was fewer compared to the reports of Kim et al. (2016) [38] on *Brassica juncea* leaves, with glucocheirolin and glucoiberverin not being detected in our study. This could be attributed to the difference in the method of extraction and the instrument used. These authors performed desulphonation during extraction and determined the desulphated GSLs, while, in this study, the glucosinolates were determined as intact glucosinolates. The indole glucosinolates, 4-hydroxyglucobrassicin, neoglucobrassicin, and 4-methoxyglucobrassicin, that have been reported in the seeds and roots of mustard [38,50] were also undetected in our study. Glucoraphasatin, the main glucosinolate of Raphanus sativus, has been reported in the roots of red mustard [50] but was undetected in our study. This could be due to the difference in the type of plant part used. The HomoMet-derived aliphatic glucosinolate, sinigrin, is highly abundant in *Brassica juncea* seeds with a concentration as high as 109.9 µmole/g DW and is showed to suppress nematode activity [11]. One of the major contributors to the total glucosinolates level in our samples, gluconapin, is also highly abundant in Chinese kale shoots (up to138.6 μmole/g DW) [16], pak choi (up to 70.67 μmole/g DW) [19], and leaf blade of Ezo-wasabi (up to 168.4 μmole/g DW) [51]. The overall glucosinolates levels in this study were lower compared to the previous reports discussed above. This could be related to a difference in sample preparation and recovery protocols. As observed previously in genotypes of mustard samples from Ethiopia [42,52], Korea [38], and different parts of the world [34,37], the glucosinolate concentrations showed a wide intra-species variation in the current study. The diversity of genetic resources reflects the frequency of important phytochemical, phenological, and morphological traits in germplasm collection, and breeding programs largely exploit the variation of these traits. The variation in the GSL content could determine important roles in the physiology, productivity, nutrition, and health benefits of mustard plants.

GSLs showed inconsistent inter- and intra-leaf variation. The spatial and stage-wise distribution of GSLs in the leaves of *Brassica juncea* could be used to explore how plants use GSLs in the defense management, ecological significance, and biosynthesis mechanisms of GSLs in Brassica plants. To the best of our knowledge, such studies are elusive in the literature. Previously, the levels of glucosinolates were found to be affected by leaf position [44] and leaf cross section [45]. Choi et al. (2014) [44] compared the level of glucosinolates between the inner and outer leaves of Brassica oleracea var. capitata in spring and fall sawing and found that the inner leaves contained higher individual glucosinolates in most of the samples. A study about the leaf spatial patterns of glucosinolate levels among different leaf regions of Raphanus sativus showed that proximal leaflets had significantly more glucosinolates compared to the main leaflet, edge, vein, and middle [45]. In a recent intra- and inter-leaf variation study on three kimchi cabbage varieties (*Brassica rapa*), Rhee et al. (2020) [20] reported that the proximal half of the leaves contained higher GSLs in most cases, that the middle layers had higher average total GSL levels compared to the inner and outer layer leaves, and suggested that the result was not strictly consistent. This experiment also found an inconsistent trend in the distribution of GSLs based on the leaf layers as well as the leaf sections.

This study is entrusted to identify potential candidates of genetic resources for different important traits for producing GSLs-dense lines and mustard leaves with high yields and other important leaf agro-morphological characters. Table 7 summarizes the promising accessions for various morphological and biochemical traits. Breeding strategies for improved glucosinolate/isothiocyanate profiles of Brassicaceae species are dependent on various factors, such as consumer preference, postharvest processing, biotic and abiotic responses, elicitor responses, and species selection [53]. The usual trend in many horticultural crops’ breeding has been mainly focused on increasing the yield, compromising the biochemical contents. Producing nutrient-dense *Brassica juncea* crops in terms of GSLs and adding them to our diet could help reduce the onset of chronic diseases such as cancer [5,54,55]. In addition, some GSLs could also contribute to producing varieties resistant to biotic and abiotic stress. Breeding crops that contain agro-ecologically relevant metabolic profiles requires investigation of the chemical diversity of large collections of germplasm. GSLs are known for their potential health benefits to humans [43,56] and as defense compounds for disease resistance in plants [57,58]. Producing lines of *Brassica juncea* with enhanced levels of GSLs requires assessment of diverse genetic resources in terms of biochemical components as well as agronomical traits. In this study, we have found that some of the genotypes highly accumulated specific GSLs. For example, the highest SIN content was recorded in IT259503 (4078.7 μmole/kg DW) and IT237840 (4184.6 μmole/kg DW), while the lowest SIN (13.0 μmole/kg DW) and the highest GNA (7708.6 μmole/kg DW) were in ‘Yeosu66’. The highest amounts of GNS and GBS were detected in IT236761 (3393.5 μmole/kg DW) and IT248036 (351.9 μmole/kg DW), respectively. The highest GIB content (93.6 μmole/kg DW), 3-fold higher than the second-highest in the entire domain, was detected in IT102894. In general, two accessions, ‘Yeosu66’ and IT259487, had relatively higher total glucosinolates (>6000 μmole/kg DW), while ‘Yeosu66’, IT237840, IT250121, IT259487, IT259503, and ‘Yeosu17’ all exhibited greater than 5000 μmole/kg DW total aliphatic glucosinolates. Given that each of the GSLs has its own significance for human health and in the plant defense mechanism, the use of these accessions in producing GSL-rich mustard lines would be highly beneficial.

Some accessions showed a superior performance on some of the agro-morphological traits. For example, IT228984 had the highest plant weight (1854.3 g), which was 1.69- and 5.47-fold higher than the second-highest and average plant weights in the whole domain. IT120115 and IT118972 were superior in leaf length, with 57 cm and 55.2 cm, respectively. Similarly, IT2288223, Yeosu42, Yeosu61, and Yeosu84 exhibited leaf widths greater than 30 cm and could be the best resources for producing wider-leaf mustards. As observed in the correlations analysis (Table 4), a wider petiole was correlated with higher levels of GNA, GBN, and GBS, while the tallness of the petiole was associated with higher levels of most GSLs. IT228223 and Yeosu80, which were also characterized by large leaf and midrib widths, could be the best germplasms in terms of width of the petiole. The superior genotypes identified for different traits in this study should also be investigated for their agro-climatic preference, disease resistance capability, and other biochemical characteristics, including their proximate compositions, antioxidant activity, and secondary metabolites, in order to be utilized in breeding programs for developing lines with enhanced yields and health benefits.

## 5. Conclusions

A total of 355 accessions collected from Korea and stored in the National Agrobiodiversity Center GenBank were evaluated for intact GSLs contents and agro-morphological characters. Four aliphatic glucosinolates (SIN, GNA, GBN, and GIB), two phenylalkyl glucosinolates, and one indole GSL were detected and determined using UPLC-MS/MS. Seventeen qualitative and eight quantitative agro-morphological characters were also evaluated. A wide variability was observed for most of the traits. This study provides enriching information for the scientific community and consumers about the agro-morphological traits and glucosinolate diversity in *B. juncea* and offers guidance for *B. juncea* selection and the development of high-quality cultivars. Promising potential candidate accessions in terms of their performance in various agro-morphological traits and GSLs levels are also identified. This report provides critical data specifically for breeders developing new varieties with enhanced levels of glucosinolates. Considering the anticancer properties of glucosinolates reported elsewhere, this report would be of interest to consumers, nutraceutical companies engaged in formulating anti-cancerous food supplements, and drug developers. Further investigation using molecular markers, disease resistance, and other agro-morphological and biochemical traits are also apt to develop high-quality leaf mustard cultivars. To the best of our knowledge, the number of accessions we used represented one of the largest sets in a single study to date.

## Figures and Tables

**Table 1:** Retention time (t_R_), MRM transitions, collision-induced dissociation values, and calibration equations of the individual GSLs identified in *Brassica juncea* leaves.

Glucosinolates	t_R_	MRM Transition	CID	Calibration Equation
Sinigrin	1.08	357.88 > 96	20	Y = 12.8988X + 16.8399, r^2^ = 0.999
Gluconapin	2.58	371.89 > 96	20	Y = 12.0411X − 13.1698, r^2^ = 0.998
Glucobrassicanapin	2.90	385.95 > 96	25	Y = 44.9459X − 37.6357, r^2^ = 0.997
Glucoiberin	1.09	421.91 > 96	20	Y = 8.48762X − 24.3857, r^2^ = 0.999
Gluconasturtiin	3.23	421.97 > 96	25	Y = 39.0356X − 44.9008, r^2^ = 0.998
Glucobarbarin	2.91	437.93 > 96	20	Y = 15.443X − 21.6364, r^2^ = 0.996
Glucobrassicin	3.01	446.95 > 96	20	Y = 28.0663X − 43.3473, r^2^ = 0.999

t_R_: Retention time; MRM: Multiple reaction monitoring; CID: Collision-induced dissociation.

**Table 2:** Modified International Union for the Protection of New Varieties of Plants (UPOV) descriptors used for the qualitative morphological characterization of *Brassica juncea*.

S/NO	Trait	Descriptions *
1	Plant habit (posture)	Erect (73), semi-erect (162), horizontal (114), mixed (6)
2	Plant: head formation	Absent (5), partially present (297), fully present (52), mixed (1)
3	Leaf attitude at the apical part	Flat (119), bend downward (215), bend upward (15), mixed (5)
4	Leaf type	Lyrate (257), division (17), entire and narrow midrib (46), entire and broad midrib (24), mixed (11)
5	Leaf shape	Lanceolate (17), oblanceolate (16), spatulate (186), ovate (67), obovate (35), elliptic (9), broad elliptic (11), compressed circular (3), mixed (11)
6	Leaf waxiness	Weak (52), medium (142), strong (148), mixed (13)
7	Lobation of leaf margins	Absent (10), lobed (103), cleft (181), parted (21), dissected (22), mixed (18)
7	Leaf blade: density of incisions of margin	Absent (8), sparse (113), medium (164), dense (29), very dense (23), mixed (18)
8	Leaf blade: size of the terminal lobe	Absent (16), small (196), medium (106), large (20), mixed (17)
9	Leaf blade: number of serrates	Absent (17), few (152), medium (127), many (47), mixed (12)
11	Leaf blade: anthocyanin coloration	Absent (27), very weak (121), weak (52), medium (65), strong (42), very strong (24), mixed (24)
12	Leaf blade: blistering	Absent/weak (80), medium (118), strong (143), mixed (14)
13	Leaf blade: pubescence on the upper side	Absent (81), present (263), mixed (11)
14	Leaf blade: pubescence on the lower side	Absent (54), few (101), medium (154), many (35), mixed (11)
15	Stem hair	Absent (59), present (291), mixed (5)
16	Midrib transection	Horizontal (41), intermediate (122), semi-circled (191)
17	Petiole anthocyanin coloration	Absent (77), present (257), mixed (18)

* Values in parentheses indicate the number of accessions in each category.

**Table 3:** Variation of quantitative morphological characters and GSLs in *Brassica juncea* leaf samples from 355 accessions.

GSLs and Agro-Morphological Characters	Range	Average ± SD	Coefficient of Variation (%)
SIN	13.0–4184.6	635.9 ± 586.4	92.2
GNA	44.1–7708.7	496.4 ± 628.5	126.6
GBN	0.1–180.2	12.8 ± 20.1	156.9
GIB	0.0–93.6	2.1 ± 6.3	299.5
GBB	0.7–33.9	4.7 ± 3.3	68.9
Total aliphatic GSLs	133.7–7738.2	1151.8 ± 982.6	85.3
GNS	27.2–3393.5	401.9 ± 342.9	85.3
GBS	17.3–351.9	57.9 ± 44.2	76.3
Total GSLs	320.4–8055.3	1610.8 ± 1064.8	66.1
Leaf length (cm)	20.8–57.0	36.3 ± 5.8	16.0
Leaf width (cm)	8.6–31.8	18.1 ± 4.3	24.0
Midrib width (cm)	0.6–3.8	1.8 ± 0.6	33.7
Midrib thickness (cm)	0.3–1.1	0.6 ± 0.1	18.9
Petiole length (cm)	0.9–12.5	3.1 ± 1.5	47.7
Petiole width (cm)	0.4–3.8	1.3 ± 0.6	48.7
Number of leaves (ea)	6.0–28.0	16.3 ± 6	36.8
Plant weight (g, FW)	91.7–1854.3	338.9 ± 152.7	45.1

The contents of glucosinolates are expressed in μmole/kg DW. Glucosinolate abbreviations: GSLs: Glucosinolates; SIN: Sinigrin; GNA: Gluconapin; GBN: Glucobrassicanapin; GIB: Glucoiberin; GBB: Glucobarbarin; GNS: Gluconasturtiin; GBS: Glucobrassicin; FW: Fresh weight; SD: Standard deviation.

**Table 4:** Pearson correlations among the glucosinolates and quantitative agro-morphological characters of 355 *B. juncea* accessions.

	Leaf Length	Leaf Width	No of Leaves	Midrib Width	Midrib Thickness	Petiole Length	Petiole Width	Plant Weight	SIN	GNA	GBN	GIB	GNS	GBB	GBS
Leaf width	0.622 **														
Number of leaves	0.303 **	0.167 **													
Midrib width	0.444 **	0.854 **	0.256 **												
Midrib thickness	0.475 **	0.581 **	0.161 **	0.455 **											
Petiole length	0.210 **	−0.128 *	−0.004	−0.229 **	0.045										
Petiole width	0.320 **	0.829 **	0.004	0.895 **	0.429 **	−0.268 **									
Plant weight	0.532 **	0.451 **	0.085	0.348 **	0.352 **	0.122 *	0.368 **								
SIN	−0.116 *	0.008	−0.009	0.051	−0.082	−0.185 **	0.055	−0.138 **							
GNA	−0.061	0.081	0.012	0.143 **	0.119 *	−0.007	0.119 *	−0.098	0.272 **						
GBN	−0.049	0.065	0.063	0.155 **	0.041	−0.166 **	0.121 *	−0.106 *	0.346 **	0.551 **					
GIB	−0.119 *	−0.005	0.038	0.04	−0.061	−0.144 **	0.061	−0.044	0.256 **	0.159 **	0.139 *				
GNS	−0.016	−0.102	0.092	−0.053	−0.087	−0.000	−0.101	−0.042	0.082	−0.085	0.097	−0.058			
GBB	−0.005	−0.009	0.012	0.048	−0.01	−0.036	0.016	−0.024	0.139 **	−0.054	0.098	0.004	0.565 **		
GBS	−0.101	0.065	−0.015	0.112 *	0.006	−0.124 *	0.127 *	−0.105 *	0.463 **	0.380 **	0.173 **	0.064	0.110 *	0.083	
Total GSLs	−0.111 *	0.024	0.032	0.103	−0.001	−0.115*	0.076	−0.154 **	0.766 **	0.740 **	0.575 **	0.226 **	0.325 **	0.235 **	0.560 **

** Correlations are significant at the 0.01 level; * Correlations are significant at the 0.05 level. Glucosinolate abbreviations: GSLs: Glucosinolates; SIN: Sinigrin; GNA: Gluconapin; GBN: Glucobrassicanapin; GIB: Glucoiberin; GBB: Glucobarbarin; GNS: Gluconasturtiin; GBS: Glucobrassicin.

**Table 5:** The correlations of agro-morphological characters with the levels of GSLs.

Morphological Characters	Descriptions (N)	SIN	GNA	GBN	GIB	GNS	GBB	GBS	Total GSLs
Plant habit (posture)	Erect (73)	**659.2** ^ab^	614.9 ^a^	12.9 ^a^	2.5 ^a^	370.4 ^a^	5.1 ^a^	**71.1** ^b^	**1735.9** ^ab^
	Semi erect (162)	**541.2** ^a^	412.3 ^a^	11.0 ^a^	1.5 ^a^	399.5 ^a^	4.5 ^a^	**48.3** ^a^	**1418.2** ^a^
	Horizontal (141)	**749.5** ^b^	537.7 ^a^	15.3 ^a^	2.7 ^a^	424.3 ^a^	4.8 ^a^	**62.8** ^b^	**1794.6** ^b^
Leaf: attitude at the apical part	Flat (119)	680.1 ^a^	535.6 ^a^	12.2 ^a^	2.6 ^a^	412.7 ^a^	4.5 ^a^	58.8 a	1703.9 ^a^
	Bend downward (215)	607 ^a^	475.1 ^a^	12.9 ^a^	1.8 ^a^	397.8 ^a^	4.9 ^a^	58.5 ^a^	1557.9 ^a^
	Bend upward (16)	674.9 ^a^	486.5 ^a^	16.8 ^a^	2.9 ^a^	406.6 ^a^	4.4 ^a^	43.9 ^a^	1635.3 ^a^
Leaf type	Lyrate (257)	**635.8** ^ab^	463.2 ^a^	12.3 ^a^	1.9 ^a^	427.2 ^a^	4.8 ^a^	**55.7** ^ab^	**1599.7** ^ab^
	Division (17)	**305.1** ^a^	431.9 ^a^	7.2 ^a^	0.3 ^a^	355.6 ^a^	4.6 ^a^	**33.1** ^a^	**1137.7** ^a^
	Entire, narrow midrib (46)	**678.5** ^ab^	631.0 ^a^	19.0 ^a^	3.2 ^a^	321.6 ^a^	4.2 ^a^	**65.8** ^bc^	**1723.1** ^ab^
	Entire, broad midrib (24)	**762.3** ^b^	668.8 ^a^	13.0 ^a^	3.9 ^a^	341.7 ^a^	5.0 ^a^	**86.2** ^c^	**1880.9** ^b^
Leaf shape	Lanceolate (17)	788.8 ^a^	400.8 ^a^	8.6 ^a^	1.7 ^a^	327.7 ^a^	4.9 ^a^	63.1 ^ab^	1595.5 ^a^
	Oblanceolate (16)	524 ^a^	343.1 ^a^	7.7 ^a^	1.3 ^a^	398.7 ^a^	4.0 ^a^	55.4 ^ab^	1334.1 ^a^
	Spatulate (186)	633.6 ^a^	467.9 ^a^	11.5 ^a^	2.1 ^a^	441.0 ^a^	4.9 ^a^	56.3 ^ab^	1615.6 ^a^
	Ovate (67)	665.5 ^a^	688.7 ^a^	20.5 ^a^	2.1 ^a^	378.8 ^a^	4.6 ^a^	57.4 ^ab^	1817.3 ^a^
	Obovate (35)	510.0 ^a^	476.4 ^a^	13.9 ^a^	1.4 ^a^	286.0 ^a^	3.6 ^a^	64.5 ^ab^	1355.7 ^a^
	Elliptic (9)	590.9 ^a^	383.8 ^a^	4.2 ^a^	2.0 ^a^	409.5 ^a^	5.0 ^a^	43.9 ^a^	1439.4 ^a^
	Broad elliptic (11)	714.1 ^a^	261.0 ^a^	4.0 ^a^	5.3 ^a^	374.2 ^a^	5.7 ^a^	65.3 ^ab^	1429.4 ^a^
	Compressed circular (3)	694.8 ^a^	676.6 ^a^	13.0 ^a^	1.5 ^a^	395.5 ^a^	7.1 ^a^	109 ^b^	1897.5 ^a^
Lobation of leaf margins	Absent (10)	600.1 ^a^	571.5 ^a^	21.1 ^b^	2.1 ^a^	298.0 ^a^	4.0 ^a^	**60.1** ^ab^	**1556.6** ^ab^
	Lobed (103)	718.9 ^a^	565.0 ^a^	16.6 ^ab^	2.4 ^a^	397.0 ^a^	4.9 ^a^	**64.2** ^ab^	**1768.9** ^ab^
	Cleft (181)	592.5 ^a^	474.7 ^a^	11.0 ^ab^	2.0 ^a^	418.2 ^a^	4.8 ^a^	**56.3** ^ab^	**1557.6** ^ab^
	Parted (21)	840.5 ^a^	668.7 ^a^	16.2 ^ab^	3.1 ^a^	387.5 ^a^	4.1 ^a^	**72.8** ^b^	**1993.0** ^b^
	Dissected (22)	416.4 ^a^	199.5 ^a^	3.6 ^a^	0.6 ^a^	351.9 ^a^	3.6 ^a^	**37.4** ^a^	**1012.8** ^a^
Leaf blade: density of incisions of margins	Absent (8)	614.3 ^a^	600.5 ^a^	**24.4** ^b^	2.2 ^a^	335.1 ^a^	4.1 ^a^	67.2 ^a^	1647.8 ^a^
	Sparse (113)	726.9 ^a^	543.3 ^a^	**16.5** ^ab^	2.3 ^a^	403.4 ^a^	4.8 ^a^	65.1 ^a^	1762.1 ^a^
	Medium (164)	591.7 ^a^	474.4 ^a^	**10.9** ^ab^	2.2 ^a^	415.0 ^a^	4.9 ^a^	55.8 ^a^	1552.8 ^a^
	Dense (29)	680.1 ^a^	557.5 ^a^	**12.9** ^ab^	2.1 ^a^	377.0 ^a^	4.2 ^a^	64.9 ^a^	1698.7 ^a^
	Very dense (23)	447.3 ^a^	351.5 ^a^	**4.3** ^a^	0.8 ^a^	355.8 ^a^	3.7 ^a^	36.2 ^a^	1199.6 ^a^
Leaf blade: size of terminal lobe	Absent (16)	755.3 ^a^	560.7 ^a^	17.9 ^b^	1.9 ^a^	296.7 ^a^	4.4 ^a^	70.5 ^a^	1707.3 ^a^
	Small (196)	623.0 ^a^	529.6 ^a^	14.2 ^ab^	2.3 ^a^	408.9 ^a^	4.6 ^a^	60.5 ^a^	1642.5 ^a^
	Medium (106)	660.9 ^a^	477.4 ^a^	11.0 ^ab^	2.1 ^a^	385.7 ^a^	4.9 ^a^	52.9 ^a^	1592.7 ^a^
	Large (20)	510.2 ^a^	253.0 ^a^	4.7 ^a^	0.7 ^a^	497.0 ^a^	5.0 ^a^	59.7 ^a^	1330.2 ^a^
Leaf blade: number of serrates	Absent (17)	906.1 ^a^	514.2 ^a^	17.4 ^a^	4.8 ^a^	373.4 ^a^	5.5 ^a^	**77.0** ^b^	1898.3 ^a^
	Few (152)	596.2 ^a^	490.8 ^a^	13.8 ^a^	1.6 ^a^	405.2 ^a^	4.7 ^a^	**55.6** ^ab^	1566.2 ^a^
	Medium (127)	670.2 ^a^	556.9 ^a^	12.6 ^a^	1.9 ^a^	394.3 ^a^	4.7 ^a^	**62.4** ^ab^	1702.9 ^a^
	Many (47)	595.6 ^a^	383.8 ^a^	9.8 ^a^	3.4 ^a^	423.6 ^a^	4.4 ^a^	**49.4** ^a^	1468.3 ^a^
Leaf blade: anthocyanin coloration	Absent (27)	453.7 ^a^	296.3 ^a^	5.6 ^a^	1.5 ^a^	320.4 ^a^	3.9 ^a^	38.5 ^a^	1119.7 ^a^
	Very weak (121)	649.0 ^a^	645.4 ^a^	16.7 ^a^	2.7 ^a^	362.0 ^a^	4.7 ^a^	64.9 ^a^	1744.8 ^a^
	Weak (52)	704.1 ^a^	410.5 ^a^	13.2 ^a^	1.6 ^a^	513.3 ^a^	5.3 ^a^	63.9 ^a^	1707.3 ^a^
	Medium (65)	742.5 ^a^	568.3 ^a^	15.4 ^a^	2.8 ^a^	367.8 ^a^	4.4 ^a^	59.3 ^a^	1760.3 ^a^
	Strong (42)	510.0 ^a^	342.0 ^a^	6.3 ^a^	1.3 ^a^	420.5 ^a^	4.5 ^a^	50.9 ^a^	1335.2 ^a^
	Very strong (24)	497.5 ^a^	342.2 ^a^	8.0 ^a^	0.5 ^a^	515.3 ^a^	5.8 ^a^	49.8 ^a^	1418.9 ^a^
Leaf waxiness	Weak (52)	651.4 ^a^	**367.6** ^a^	**11.3** ^ab^	1.1 ^a^	449.4 ^a^	4.4 ^a^	**63.6** ^b^	**1548.6** ^ab^
	Medium (142)	528.9 ^a^	**417.0** ^ab^	**9.6** ^a^	2.0 ^a^	377.2 ^a^	4.6 ^a^	**45.5** ^a^	**1382.4** ^a^
	Strong (148)	721.9 ^a^	**619.7** ^b^	**16.6** ^b^	2.5 ^a^	410.1 ^a^	5.0 ^a^	**68.9** ^b^	**1844.6** ^b^
Leaf blade: blistering	Absent/weak (80)	663.2 ^a^	465.3 ^a^	13.4 ^a^	1.9 ^a^	415.5 ^a^	4.6 ^a^	61.9 ^a^	1625.8 ^a^
	Medium (118)	590.0 ^a^	518.3 ^a^	13.1 ^a^	1.7 ^a^	368.9 ^a^	4.9 ^a^	57.5 ^a^	1552.3 ^a^
	Strong (143)	647.6 ^a^	496.0 ^a^	12.2 ^a^	2.5 ^a^	419.8 ^a^	4.6 ^a^	57.1 ^a^	1639.1 ^a^
Leaf blade: pubescence on the upper side	Absent (81)	668.8	653.7	20.1	2.8	319.9	4.9	68.8	1738.9
	Present (263)	633.5	449.3	10.8	1.9	427.2	4.7	55.3	1581.5
Leaf blade: pubescence on the lower side	Absent (54)	**677.9** ^b^	**661.5** ^b^	**20.5** ^b^	3.2 ^a^	310.7 ^a^	5.0 ^a^	**74.1** ^b^	**1752.8** ^b^
	Few (101)	**687.0** ^b^	**483.3** ^ab^	**14.2** ^ab^	1.7 ^a^	385.5 ^a^	4.9 ^a^	**61.2** ^b^	**1637.7** ^ab^
	Medium (154)	**659.5** ^b^	**478.9** ^ab^	**11.0** ^a^	2.4 ^a^	449.0 ^a^	4.8 ^a^	**55.9** ^ab^	**1659.3** ^ab^
	Many (35)	**378.1** ^a^	**366.5** ^a^	**7.2** ^a^	0.6 ^a^	383.0 ^a^	3.7 ^a^	**37.9** ^a^	**1177.0** ^a^
Stem hair	Absent (59)	670.9	652.4	20.2	3.2	321.2	5.0	73.8	1746.5
	Present (291)	630.8	461.0	11.4	1.9	417.9	4.7	55.0	1581.6
Midrib transection	Horizontal (41)	**837.8** ^b^	**684.4** ^b^	**22.8** ^b^	2.4 ^a^	434.0 ^a^	5.2 ^a^	**73.1** ^b^	**2059.7** ^b^
	Intermediate (122)	**589.1** ^a^	**541.8** ^ab^	**12.8** ^a^	2.0 ^a^	420.8 ^a^	4.8 ^a^	**55.3** ^a^	**1626.6** ^a^
	Semi-circled (191)	**604.4** ^a^	**422.8** ^a^	**10.6** ^a^	2.0 ^a^	383.3 ^a^	4.6 ^a^	**56.2** ^a^	**1482.1** ^a^
Petiole anthocyanin coloration	Absent (77)	468.6	418.8	8.7	1.0	385.1	4.3	43.2	1329.7
	Present (259)	683.5	531.9	14.4	2.5	411.9	4.8	62.7	1710.5
Plant head formation	Absent (5)	263.5 ^a^	216.4 ^a^	5.5 ^a^	0.2 ^a^	326.0 ^a^	3.5 ^a^	**30.9** ^a^	846.0 ^a^
	Partially present (297)	635.1 ^a^	483.5 ^a^	12.6 ^a^	2.0 ^a^	421.4 ^a^	4.8 ^a^	**55.7** ^ab^	1613.9 ^a^
	Fully present (52)	697.7 ^a^	619.4 ^a^	15.6 ^a^	3.1 ^a^	301.8 ^a^	4.5 ^a^	**75.0** ^b^	1716.9 ^a^

N = number of accessions. Glucosinolate abbreviations: GSLs: Glucosinolates; SIN: Sinigrin; GNA: Gluconapin; GBN: Glucobrassicanapin; GIB: Glucoiberin; GBB: Glucobarbarin; GNS: Gluconasturtiin; GBS: Glucobrassicin. Different letters within the same column and the same morphological characters denote significant differences (*p* < 0.05). Values in parentheses indicate the number of accessions in each category.

**Table 6:** Eigenvalues, proportions of variability, and agro-morphological traits contributed to the first five principal components of *Brassica juncea* genetic resources.

Traits	PC1	PC2	PC3	PC4	PC5
Leaf length	0.36066	0.42513	0.050563	−0.12065	−0.014741
Leaf width	0.48364	−0.08839	−0.053683	0.099252	0.13102
No of leaves	0.14067	0.23187	0.90222	−0.06798	0.070513
Midrib width	0.45814	−0.24269	0.090063	0.072238	0.3238
Midrib thickness	0.35029	0.16403	−0.055367	0.54633	−0.70066
Petiole length	−0.055534	0.67376	−0.25685	0.37261	0.51955
Petiole width	0.43236	−0.34385	−0.16308	0.053491	0.27198
Plant weight	0.30598	0.31576	−0.27717	−0.72497	−0.19486
Eigenvalue	3.78061	1.43425	0.997487	0.572543	0.398334
% Variance	47.258	17.928	12.469	7.1568	4.9792

**Table 7:** Top ten best accessions identified for different agro-morphological and biochemical traits.

S/No	Traits	Top Ten Accessions *
1	Leaf length (≥50 cm)	IT120115, IT118972, IT118979, IT203623, IT228987, IT102888, Yesou86, IT215792, and IT120114
2	Leaf width (≥28 cm)	Yeosu84, Yeosu 42, Yeosu61, IT228223, IT204152, Yeosu27, Yeosu 57, IT203623, Yeosu26, and Yeosu20
3	No of leaves per plant (≥26 leaves)	IT228222, IT204153, IT215792, IT218388, IT235416, IT216873, IT118948, IT235418, IT180994, IT235344, IT216872, IT218451, IT218458, IT118955, and IT218449
4	Midrib width (≥3.15 cm)	Yeosu80, IT228223, IT203623, Yeosu57, Yeosu15, IT204152, Yeosu84, IT228990, IT228988, and Yeosu61
5	Midrib thickness (≥0.75 cm)	IT218357, Yeosu42, Yeosu27, IT141424, Yeosu41, IT102888, IT208801, Yeosu6, Yeosu15, IT118974, Yeosu11, and IT259499
6	Petiole length (≥6.3 cm)	IT102941, IT102942, IT259517, IT100949, IT141423, IT228987, IT259499, IT100945, IT218383, and IT141422
7	Petiole width (≥2.9 cm)	Yeosu57, Yeosu80, IT228223, Yeosu61, IT191096, IT228988, IT228988, IT204152, Yeosu21, and Yeosu24
8	Plant weight (≥650 g)	IT228984, Yeosu45, IT118979, IT215792, IT228223, IT100949, IT248039, Yeosu42, Yeosu64, IT228987
9	Sinigrin (≥2295)	IT237840, IT259503, IT248036, IT259487, IT236762, IT248037, IT250121, IT247853, IT235418, and Yeosu17
10	Gluconapin (≥2000)	Yeosu66, Yeosu31, IT102941, Yeosu17, IT218460, IT191097, Yeosu41, Yeosu32, Yeosu2, IT250121, and IT259487
11	Glucobrassicanapin (≥70)	Yeosu31, Yeosu37, Yeosu53, Yeosu41, Yeosu23, Yeosu17, Yeosu32, Yeosu44, Yeosu52, and Yeosu75
12	Glucoiberin (≥16)	IT102894, Yeosu74, Yeosu2, IT259503, IT250121, Yeosu63, Yeosu52, IT259514, IT259492, and Yeosu38
13	Gluconasturtiin (≥1340)	IT236761, IT248035, IT259491, IT248614, IT237839, IT235624, IT235803, Yeosu16, IT259513, and Yeosu23
14	Glucobarbarin (≥13)	IT102937, IT102950, IT236761, IT109154, IT218453, IT248035, Yeosu43, IT235415, Yeosu80, and IT237839
15	Glucobrassicin (≥180)	IT248036, IT248037, Yeosu66, Yeosu20, Yeosu19, Yeosu80, Yeosu21, IT259487, Yeosu18, and IT259509
16	Total Glucosinolates (≥4600)	Yeosu66, IT259487, IT259503, IT237840, Yeosu17, IT248036, IT248035, IT250121, IT248037, and Yeosu31

* Samples are arranged in decreasing order of their values; the GSLs levels are reported in μmole/kg DW.

## Data Availability

The datasets generated for this study are contained within the article.
